# Estimativa do Diâmetro da Via de Saída do Ventrículo Esquerdo: Proposta de uma Equação para Uso Clínico em Adultos a Partir dos Dados do Estudo ELSA-Brasil

**DOI:** 10.36660/abc.20260036

**Published:** 2026-09-10

**Authors:** Giulia Bevilacqua Schmitz, Bruce Bartholow Duncan, Murilo Foppa, Angela Barreto Santiago Santos

**Affiliations:** 1 Pós-Graduação em Cardiologia e Ciências Cardiovasculares Faculdade de Medicina Universidade Federal do Rio Grande do Sul Porto Alegre RS Brasil Pós-Graduação em Cardiologia e Ciências Cardiovasculares - Faculdade de Medicina - Universidade Federal do Rio Grande do Sul, Porto Alegre, RS – Brasil; 2 Hospital de Clínicas de Porto Alegre Porto Alegre RS Brasil Serviço de Cardiologia, Hospital de Clínicas de Porto Alegre, Porto Alegre, RS – Brasil; 3 Pós-Graduação em Epidemiologia Universidade Federal do Rio Grande do Sul Porto Alegre RS Brasil Pós-Graduação em Epidemiologia, Universidade Federal do Rio Grande do Sul, Porto Alegre, RS – Brasil

**Keywords:** Estenose da Valva Aórtica, Ecocardiografia, Cuidados Críticos

## Abstract

O diâmetro da via de saída do ventrículo esquerdo (DVSVE) é essencial para estimar o volume sistólico do ventrículo esquerdo e a gravidade da estenose aórtica, mas limitações técnicas podem afetar sua precisão. Um DVSVE predito pode superar esses problemas, embora ainda não tenha sido validado em grandes populações. Estudamos os participantes da amostra aleatória representativa com idade ≥ 60 anos do estudo Elsa-Brasil que realizaram ecocardiograma (n=3.267), divididos em um subgrupo de derivação (70%) e um subgrupo de validação (30%). Na coorte de derivação, foram identificados determinantes independentes do DVSVE e derivada uma equação de regressão. Na coorte de validação, o desempenho do VSVE_ELSA_ foi avaliado e comparado com fórmulas publicadas anteriormente. Na nossa amostra de derivação (n = 1.822; 58,9 ± 9,5 anos; 52% mulheres), o DVSVE médio medido foi de 19,5 ± 2,5 mm. Os preditores mais fortes do DVSVE foram sexo, altura, diâmetro da raiz da aorta (AoDiam) e diâmetro diastólico final do ventrículo esquerdo (DDVE), resultando na equação VSVE_ELSA_ = 0,03 + [0,59 x *sexo masculino*] + [0,04 x *altura*] + [0,22 x *AoDiam*] + [0,13 x DDVE]. Na amostra de validação (n = 739), o VSVE_ELSA_ apresentou alta correlação (r = 0,63) com o DVSVE medido, com concordância semelhante à do VSVE_Pestelli_ (r = 0,61), porém inferior à do VSVE_SC_ (r = 0,47). Essas diferenças se refletem em diferenças relevantes nos volumes sistólicos. O VSVE_ELSA_ apresentou bom desempenho em uma população adulta em geral e pode ser usado como alternativa quando a medição ecocardiográfica direta do DVSVE não for viável.

## Introdução

A medição do diâmetro da via de saída do ventrículo esquerdo (DVSVE) por ecocardiografia é fundamental para a estimativa do débito cardíaco^1^que pode ser útil no manejo de pacientes em estado crítico,^2^ e para classificar a gravidade da estenose aórtica. No entanto, limitações técnicas podem prejudicar a viabilidade e a precisão dessa medição. Dois estudos anteriores, incluindo participantes com estenose aórtica e insuficiência cardíaca, encontraram que a área de superfície corporal (SC), a raiz da aorta, o diâmetro do ventrículo esquerdo (VE) e outros dados antropométricos podem prever o DVSVE.^3,4^

Com base no estudo ELSA-Brasil,^5,6^ o presente estudo teve como objetivo identificar preditores de DVSVE e derivar uma equação para predizer esta medida (VSVE_ELSA_); validar o desempenho da VSVE_ELSA_ em relação a outras equações publicadas^3,4^ em uma amostra de validação mais ampla e estimar seu impacto no volume sistólico (VS) do VE derivado.

## Métodos

Os participantes foram submetidos a ecocardiograma transtorácico (ETT), entre 2008 e 2010, com base em diretrizes publicadas. Estudamos os participantes da amostra aleatória representativa com idade ≥ 60 anos do estudo Elsa-Brasil, que realizaram ecocardiograma (n=3.267), divididos em um subgrupo de derivação (70%) e um subgrupo de validação (30%). Na coorte de derivação, análises de regressão univariada e multivariada identificaram determinantes independentes do DVSVE e geraram uma equação preditiva. Para validá-la, testamos o desempenho do VSVE_ELSA,_ e de outras fórmulas publicadas (VSVE_Pestelli_ e VSVE_SC_) no subgrupo de validação. O DVSVE foi medido na mesossístole, de borda interna a borda interna, a partir de uma janela paraesternal longitudinal 2D focada. Calculamos o DVSVE estimado a partir de fórmulas previamente publicadas^3,4^ e usando a fórmula de Mosteller.^7^

## Resultados

A coorte de derivação incluiu 1.822 participantes, dos quais 52,1% eram mulheres (n=950) e 54,9% (n=1.001) se autodeclararam brancos. A prevalência de hipertensão foi de 46,5%, tabagismo de 10%, diabetes de 21,8%, sobrepeso de 42,2%, obesidade de 20,5% e doença arterial coronariana de 6,3%. O diâmetro médio da VSVE medido foi de 19,5 ± 2,5 mm, com diferenças significativas entre os sexos após ajuste por altura (masculino: 0,12±0,01 e feminino: 0,11 ± 0,01; p<0,005), mas não quando ajustado pela SC (masculino: 11,02 ± 1,4 e feminino: 10,95 ± 1,4; p=0,22).

A Tabela 1 mostra as análises univariadas e multivariadas, a partir das quais derivamos a equação para prever o DVSVE:

**Tabela 1:** – Análise de regressão linear univariada e multivariada com DVSVE medido na amostra de derivação

n=1.822	Univariado		Multivariável	
	Coeficiente b	p	Coeficiente b	p
Idade	-0,10	<0,0001		
Masculino	0,48	<0,0001	0,13	<0,0001
SC	0,44	<0,0001	0,03	0,31
Altura	0,48	<0,0001	0,13	<0,0001
Pressão arterial sistólica	0,05	0,01		
Pressão arterial diastólica	0,07	0,001		
Raiz da aorta	0,53	<0,0001	0,33	<0,0001
Diâmetro diastólico do VE	0,46	<0,0001	0,28	<0,0001
Volume diastólico do VE	0,45	<0,0001		
Volume sistólico do VE	0,38	<0,0001		
Massa do VE	0,39	<0,0001	-0,07	0,01
Fração de ejeção do VE %	-0,22	<0,0001		

SC: área de superfície corporal; PA: pressão arterial; VE: ventrículo esquerdo.


$${\text{ LVOT }}_{\text{ELSA }}=0,03+[0,59\times \text{ masculino }]+[0,04\times \text{ altura }]+[0,22\times \text{ AoDiam }]+[0,13\times \text{ DDVE }]$$

*Onde: AoDiam: diâmetro da raiz da aorta (mm); DDVE: diâmetro diastólico final do ventrículo esquerdo (mm).*

A coorte de validação consistiu em 739 participantes com características demográficas e ecocardiográficas semelhantes às da amostra de derivação. O DVSVE médio medido na amostra de validação foi de 19,4 ± 2,4 mm. O VSVE_ELSA_ apresentou alta correlação com o DVSVE medido (r = 0,63), valor próximo ao observado com o VSVE_Pestelli_ (r = 0,61) e superior ao do VSVE_SC_ (r = 0,47). Além disso, o VSVE_ELSA_ apresentou menor diferença média em relação ao DVSVE medido quando comparado ao VSVE_Pestelli_ e ao VSVE_SC_, conforme demonstrado na Tabela 2. Ademais, o VS apresentou resultados semelhantes (Tabela 2). Todos os resultados estão apresentados na Figura 1.

**Tabela 2:** – Comparação de diferentes fórmulas para o DVSVE e o VS do VE previstos na amostra de validação

	Média ±DP	Correlação de Pearson com VSVE	Diferença média com VSVE (média ± DP)	P para a diferença com o VSVE medido
VSVE (n=739)				
DVSVE medido, mm	19,4 ± 2,4			
VSVE_ELSA_, mm	19,4 ± 1,7	0,63*	-0,03 ± 1,85	p=0,58
VSVE_Pestelli_, mm	21,0 ± 1,2	0,61*	-1,60 ± 1,90	p<0,0001
VSVE_SC_, mm	22,3 ± 1,1	0,47*	-2,84 ± 2,11	p<0,0001
Volume Sistólico (n=739)				
VS calculado, ml/batimento	58,4 ± 16,9			
VS VSVE_ELSA_, ml/batimento	58,3 ± 15,6	0,74*	-0,21 ± 11,76	p=0,63
VS VSVE_Pestelli_, ml/batimento	67,9 ±16,1	0,70*	-9,83 ± 12,60	p<0,0001
VS VSVE_SC_, ml/batimento	76,8 ±18,1	0,65*	-18,42 ± 14,54	p<0,0001

DVSVE: diâmetro da via de saída do ventrículo esquerdo; VS: volume sistólico. *p<0,0001.

**Figura 1:**
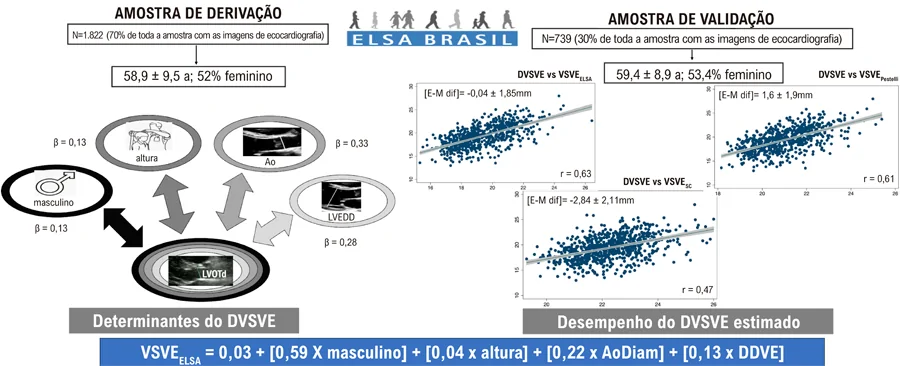
– Diâmetro estimado da VSVE: proposta de uma equação para uso clínico em adultos a partir dos dados do Estudo ELSA Brasil.

## Discussão

Está bem estabelecido que as medidas ecocardiográficas são influenciadas por fatores demográficos, como medidas antropométricas (altura, peso e área de superfície corporal), sexo e idade.^8^Um estudo de Leye et al., em um grupo de pacientes com e sem estenose aórtica, mostrou que o DVSVE era significativamente maior em homens em comparação com mulheres. Essa diferença persistiu após o ajuste para altura, mas não após a correção da SC, similar aos nossos resultados.^3^Em uma população com insuficiência cardíaca, Pestelli et al.^4^ acharam que o AoDiam e o DDVE afetam o DVSVE independentemente dos dados antropométricos. A inclusão desses dados na equação derivada para prever o DVSVE permitiu que eles encontrassem uma alta correlação com o DVSVE medido e um melhor desempenho em comparação com a equação baseada na SC.

Reconhecemos algumas limitações em nosso estudo. Nossa equação é derivada de quatro variáveis, duas das quais são clinicamente plausíveis e facilmente obtidas a partir de parâmetros ecocardiográficos de rotina, embora dependam da qualidade da imagem e da aquisição pelo operador. Além disso, nossa amostra não foi validada por outros métodos de imagem ou em populações específicas. Pode-se observar, no entanto, que a equação proposta pode não ser apropriada para valores extremos de DVSVE.

Em conclusão, constatamos que as variáveis antropométricas (sexo e altura) e ecocardiográficas (AoDiam e DDVE) foram os principais preditores do diâmetro da via de saída do ventrículo esquerdo em uma população adulta geral, o que está de acordo com os achados de fórmulas anteriores desenvolvidas em coortes específicas para determinadas doenças. A forte associação entre o DVSVE medido e o estimado pela equação VSVE_ELSA_ pode reforçar a validade clínica de uma ferramenta complementar quando a medição direta não é viável.
