# Supplementary material for: Estimativa do Diâmetro da Via de Saída do Ventrículo Esquerdo: Proposta de uma Equação para Uso Clínico em Adultos a Partir dos Dados do Estudo ELSA-Brasil
Source: Arq Bras Cardiol. 2026 Sep 10;123(8):e20260036. [Article in Portuguese] doi: 10.36660/abc.20260036 (PMC13671728; doi:10.36660/abc.20260036)
Supplement: Translation

# Translation: Estimated Left Ventricular Outflow Tract Diameter: Proposal of an Equation for Clinical Use in Adults from The ELSA Brasil Study data

**DOI:** 10.36660/abc.20260036i

**Authors:** Giulia Bevilacqua Schmitz, Bruce Bartholow Duncan, Murilo Foppa, Angela Barreto Santiago Santos

**Affiliations:** 1 Brazil Post-Graduate Program in Cardiology and Cardiovascular Sciences, Medical School, Universidade Federal do Rio Grande do Sul, Porto Alegre, RS – Brazil; 2 Brazil Cardiology Division, Hospital de Clínicas de Porto Alegre, Porto Alegre, RS – Brazil; 3 Brazil Post-Graduate Program in Epidemiology, Universidade Federal do Rio Grande do Sul, Porto Alegre, RS – Brazil

**Keywords:** Aortic Valve Stenosis, Echocardiography, Critical Care

## Abstract

Left ventricular outflow tract diameter (LVOTd) is essential for estimating stroke volume and aortic stenosis severity, but technical limitations may affect its accuracy. A predicted LVOTd may overcome these issues, though it has not been validated in large populations. We studied the participants of the representative random sample and with ≥ 60 y-o from the Elsa-Brasil study with echocardiogram performed (n=3,267), divided into a derivation (70%) and a validation subgroup (30%). In the derivation cohort, independent determinants of LVOTd were identified, and a regression equation was derived. In the validation cohort, the performance of LVOT_ELSA_ was evaluated and compared with previously published formulas. In our derivation sample (n= 1,822; 58.9 ± 9.5 y-o; 52% females), the mean measured LVOTd was 19.5 ± 2.5 mm. The strongest predictors of LVOTd were sex, height, aortic root diameter (AoDiam), and LV end-diastolic diameter (LVEDD), deriving the equation LVOT_ELSA_ = 0.03 + [0.59 x *male*] + [0.04 x *height*] + [0.22 x *AoDiam*] + [0.13 x *LVEDD*]. In the validation sample (n=739), LVOT_ELSA_ showed a high correlation (r=0.63) with measured LVOTd with similar agreement to LVOT_Pestelli_ (r=0.61), but worse with LVOT_BSA_(r=0.47). These differences are reflected in relevant differences in stroke volumes. LVOT_ELSA_ showed good performance in a general adult population and may be used as an alternative when direct echocardiographic measurement of LVOTd is not feasible.

## Introduction

The measurement of left ventricular outflow tract diameter (LVOTd) by echocardiography is a cornerstone for the estimation of cardiac output^1^ which may be useful in the management of critically ill patients,^2^ and for grading the severity of aortic stenosis. However, technical limitations may impair the feasibility and accuracy of this measurement. Two previous studies, including participants with aortic stenosis and heart failure, have found that body surface area (BSA), aortic root, left ventricular (LV) diameter, and other anthropometric data can predict LVOTd.^3,4^

Based on the ELSA-Brasil study^5,6^ the present study aimed to identify LVOTd predictors and derive an equation to predict them (LVOT_ELSA_); to validate the performance of LVOT_ELSA_ related to other published equations^3,4^ in a wider validation sample, and to estimate its impact on the echocardiographically derived LV stroke volume (SV).

## Methods

Participants underwent standardized transthoracic echocardiogram (TTE) between 2008 and 2010 based on published guidelines. We studied the participants of the representative random sample and with ≥ 60 y-o from the Elsa-Brasil study with echocardiogram performed (n=3,267), divided into a derivation (70% of these) and a validation subgroup (30%). In the derivation cohort, univariate and multivariable regression analyses identified independent LVOTd determinants and generated a predictive equation. To validate it, we tested the performance of the LVOT_ELSA,_ and of other published formulas (LVOT_Pestelli_, and LVOT_BSA_) in the validation subgroup. LVOTd was measured in mid-systole using the inner edge to inner edge, from a focused 2D parasternal long-axis view. We calculated the estimated LVOTd from previously published formulas^3,4^ and using the Mosteller formula.^7^

## Results

The derivation cohort included 1,822 participants, of whom 52.1% were female (n=950) and 54.9% (n=1,001) self-reported as white. The prevalence of hypertension was 46.5%, smoking 10%, diabetes 21.8%, overweight 42.2%, obesity 20.5%, and coronary heart disease 6.3%. The mean measured LVOTd was 19.5 ± 2.5 mm, with significant differences by sex when adjusted by height (male: 0.12 ± 0.01, and female: 0.11 ± 0.01; p<0.005), but not when adjusted by BSA (male: 11.02 ± 1.4, and female: 10.95 ± 1.4; p=0.22).

Table 1 shows the univariate and multivariate analyses, from which we derived the equation for predicting LVOTd as:

**Table 1:** – Univariate and multivariable linear regression analysis with measured LVOTd in the derivation sample

n=1,822	Univariate		Multivariable	
	b coefficient	p	b coefficient	p
Age	-0.10	<0.0001		
Male	0.48	<0.0001	0.13	<0.0001
BSA	0.44	<0.0001	0.03	0.31
Height	0.48	<0.0001	0.13	<0.0001
Systolic BP	0.05	0.01		
Diastolic BP	0.07	0.001		
Aortic root	0.53	<0.0001	0.33	<0.0001
LV diastolic diameter	0.46	<0.0001	0.28	<0.0001
LV diastolic volume	0.45	<0.0001		
LV systolic volume	0.38	<0.0001		
LV mass	0.39	<0.0001	-0.07	0.01
LV ejection fraction %	-0.22	<0.0001		

BSA: body surface area; BP: blood pressure; LV: left ventricle.


 LVOTELSA =0.03+[0.59× male ]+[0.04× height ]+[0.22× AoDiam ]+[0.13× LVEDD ]

Where: Ht: height (cm); AoDiam: aortic root diameter (mm); LVEDD: Left ventricular end-diastolic diameter (mm).

The validation cohort consisted of 739 participants with demographic and echocardiographic characteristics similar to those of the derivation sample. The mean measured LVOTd in the validation sample was 19.4 ± 2.4 mm. LVOT_ELSA_ showed a high correlation with measured LVOTd (r = 0.63), which was close to that found with LVOT_Pestelli_ (r = 0.61), and better than that of LVOT_BSA_ (r = 0.47). Additionally, LVOT_ELSA_ showed a lower mean difference in measured LVOTd when compared to LVOT_Pestelli_ and LVOT_BSA_, as demonstrated in Table 2. Furthermore, SV presented similar results (Table 2). All results are presented in Figure 1.

**Table 2:** – Comparison of different formulas for predicted LVOT diameter and SV in the validation sample.

	Mean ±SD	Pearson’s correlation with LVOT	Mean difference with LVOT (mean ± SD)	P for difference with measured LVOT
LVOT (n=739)				
LVOTd measured, mm	19.4 ± 2.4			
LVOT_ELSA_, mm	19.4 ± 1.7	0.63*	-0.03 ± 1.85	p=0.58
LVOT_Pestelli_, mm	21.0 ± 1.2	0.61*	-1.60 ± 1.90	p<0.0001
LVOT_BSA_, mm	22.3 ± 1.1	0.47*	-2.84 ± 2.11	p<0.0001
Stroke Volume (n=739)				
SV calculated, ml/beat	58.4 ± 16.9			
SV LVOT_ELSA_, ml/beat	58.3 ± 15.6	0.74*	-0.21 ± 11.76	p=0.63
SV LVOT_Pestelli_, ml/beat	67.9 ±16.1	0.70*	-9.83 ± 12.60	p<0.0001
SV LVOT_BSA_, ml/beat	76.8 ±18.1	0.65*	-18.42 ± 14.54	p<0.0001

LVOTd: left ventricular outflow tract diameter; SV: stroke volume. *p<0.0001.

**Figure 1:** – Estimated LVOTd: proposal of an equation for clinical use in adults from The ELSA Brasil Study data.

## Discussion

It has been well established that echocardiographic measurements are influenced by demographic factors such as anthropometric measures (height, weight, and BSA), sex, and age.^8^ A study by Leye et al, in a set of patients with and without aortic stenosis, showed that LVOTd was significantly larger in males compared to females. This difference remained after height adjustment, but not after BSA correction, similar to our results.^3^ In a heart failure population, Pestelli et al.^4^ found that AoDiam and LVEDD affect LVOTd independently from anthropometric data. The inclusion of these data in the derivated equation for predicting LVOTd allowed them to find a high correlation with LVOTd measured and better performance compared with the BSA-equation.

We acknowledge some limitations in our study. Our equation is derived from four variables, two of which are clinically plausible and easily obtained from routine echocardiographic parameters, although they depend on image quality and operator acquisition. Furthermore, our sample has not been validated by other imaging methods or in particular populations. It could be noticed, however, that the proposed equation may not be appropriate for the extreme values of LVOTd.

In conclusion, we found that anthropometric (sex and height) and echocardiographic variables (AoDiam and LVEDD) were the main predictors of the left ventricular outflow tract diameter in a general adult population, consistent with findings from prior formulas developed in disease-specific cohorts. The strong association of measured LVOTd and that estimated from the LVOT_ELSA_ equation may reinforce the clinical validity of an adjunctive tool when direct measurement is not feasible.
